# Objective measurement of tummy time in infants (0-6 months): A validation study

**DOI:** 10.1371/journal.pone.0210977

**Published:** 2019-02-27

**Authors:** Lyndel Hewitt, Rebecca M. Stanley, Dylan Cliff, Anthony D. Okely

**Affiliations:** Early Start, Faculty of Social Sciences and Illawarra Health and Medical Research Institute, University of Wollongong, Northfields Avenue, Wollongong, New South Wales, Australia; University of Illinois at Urbana-Champaign, UNITED STATES

## Abstract

The 2017 Australian and Canadian 24-hour movement guidelines recommend infants receive 30 minutes of tummy time daily. Currently, there are no validated objective measurement tools or devices to assess tummy time. The purpose of this study was to: 1) test the practicality of using devices on infants as an objective measure of tummy time, and 2) test the accuracy of developed algorithms and cut-points for predicting prone posture. Thirty-two healthy infants aged 4 to 25 weeks completed a protocol of 12 positions. Infants were placed in each position for 3 minutes while wearing a MonBaby (chest), GENEActiv (right hip) and two ActiGraphs (right hip and ankle). Direct observation was the criterion measure. The accuracy of the algorithms or cut-points to predict prone on floor, non-prone and prone supported positions were analyzed. Parents also completed a practicality questionnaire. Algorithms and cut-points to classify posture using devices from MonBaby, GENEActiv and ActiGraph (hip and ankle) were 79%, 95%, 90% and 88% accurate at defining tummy time and 100%, 98%, 100% and 96% accurate at defining non-prone positions, respectively. GENEActiv had the smallest mean difference and limits of agreement (-8.4s, limits of agreement [LoA]: -78.2 to 61.3s) for the prone on floor positions and ActiGraph Hip had the smallest mean difference and LoA for the non-prone positions (-0.2s, LoA: -1.2 to 0.9s). The majority of parents agreed all devices were practical and feasible to use with MonBaby being the preferred device. The evaluated algorithms and cut-points for GENEActiv and ActiGraph (hip) are of acceptable accuracy to objectively measure tummy time (time spent prone on floor). Accurate measurement of infant positioning practices will be important in the observation of 24-hour movement guidelines in the early years.

## Introduction

The Australian and Canadian 24-Hour Movement Guidelines for the Early Years (Birth to 5 years) recommend infants (Birth to one year) participate in supervised, interactive floor-based play in safe environments [1, 2]. Tummy time, defined as awake time in the prone position [3] is advantageous for motor development [4]. Thirty minutes of tummy time, spread throughout the day whilst the infant is awake and supervised is encouraged [5]. In addition, a lack of tummy time is one of the most commonly reported risk factors for the development of deformational plagiocephaly [6]. Despite this, evidence remains inconsistent, with some studies reporting a significant positive effect of tummy time on plagiocephaly and others reporting no effect [7–12].

Current research investigating tummy time has used retrospective parent questionnaires [13–16] or diaries to record infant positioning over a specified period of time [17]. These studies do not report the validity of the questionnaires used and are based on parent recall. The parents’ accuracy for estimating the time an infant spends in different activities is unclear [18] and may result in a bias towards over-reporting tummy time [19]. To enable an objective measure of the amount of tummy time an infant engages in requires the development and validation of measurement devices that can calculate real-time infant positioning [20].

A number of commercial devices are currently available that have the capacity to measure infant body posture. MonBaby is a digital baby monitor that uses a wireless connection via Bluetooth Low Energy (BLE) to an iPhone or Android phone (MonDevices Inc, New York, NY, USA). MonBaby has been originally designed to function as a “Smart Breathing Movement Monitor for Babies” (www.monbaby.com). It consists of a device (“Smart button”), which sends information about the baby’s breathing movement, body position (supine or prone), fall detection, proximity to the phone, battery life and connection status to a MonBaby app that has been installed on an iPhone or Android phone. The ActiGraph and GENEActiv devices are tri-axial accelerometry-based activity monitors (ActiGraph LLC, Pensacola, FL, USA; Activinsights Ltd, Cambridgeshire, UK). They have been successfully used in studies to assess physical activity in ambulating children [21, 22]. Whilst these devices have the capacity to measure body posture or movement, they have not been validated to measure posture in the infant population. It is also unclear where to best place a device on an infant’s body to enable this type of measurement to occur [23]. Therefore, the purpose of this study was to: 1) test the practicality of using devices on infants as an objective measure of tummy time, and 2) test the accuracy of the manufacturer’s (MonDevices Inc, ActiGraph LLC and Activinsights Ltd) algorithm or cut-points for predicting posture.

## Materials and methods

### Participants

A convenience sample of 32 infants aged from 4 to 25 weeks (male, N = 19) were recruited from the Illawarra region of New South Wales, Australia. Most infants were recruited from their Early Childhood Nurse or from advertisements within the University of Wollongong. The study was approved by the University of Wollongong and Illawarra Shoalhaven Local Health District Health and Medical Human Research Ethics Committee (HE16/167). Written informed consent was obtained by the infant’s parent prior to commencement of the study.

### Procedures

**Fig 1:**
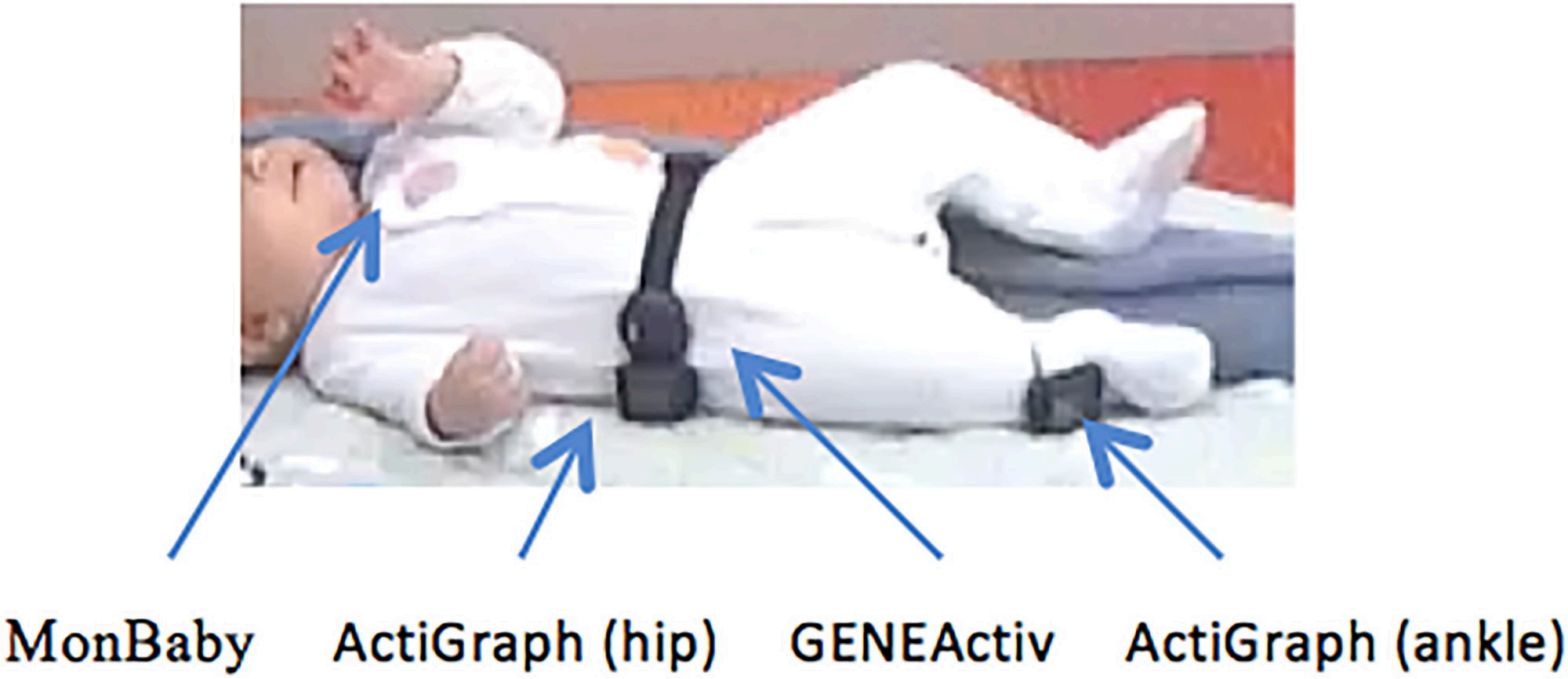
Device placement on infants. 14-week-old female wearing four devices as indicated. The parent of the individual in this manuscript has given written informed consent (as outlined in PLOS consent form) to publish these case details.

The parent brought the infant to the University of Wollongong between September 2016 and November 2017. Weight and recumbent length were measured to the nearest 0.01 kg and 0.5 cm respectively using the Seca 334 electronic baby scale plus Seca 232 measuring rod [24]. Four devices were placed onto the infant. An ActiGraph and GENEActiv were placed on the right hip (secured with an elastic strap around the waist), an ActiGraph was placed on their right ankle (secured with an elastic strap around the ankle) and a MonBaby was secured to the clothing in the middle of the infant’s chest (Fig 1). All monitors were worn continuously during testing (approximately 1 hour). Times (video and four devices) were synchronized immediately before testing. Placement of the monitors on the hip and chest were chosen based on the respective manufacturer’s recommendation. An additional ActiGraph was placed on the ankle because it is one of the most common monitoring sites in infant sleep studies [25]. Activity trials were conducted firstly on a doll, then on randomly selected infants to assist with selecting the appropriate threshold for each axis or developing an algorithm to deduce position.

The participants were placed in 12 positions by their parent: 1. prone on floor attempt one and 2. prone on floor attempt two (categorized as prone on floor positions); 3. supine, 4. left sidelying, 5. right sidelying, 6. cradle hold, 7. reclined in a car seat, 8. being held upright against the parent’s shoulder whilst the parent is standing, 9. supported sitting on the lap of the parent, and 10. reclined in a pram (categorized as non-prone positions); 11. being held whilst the infant is on their tummy (carer standing or sitting) and 12. prone but lying on the parent’s chest who was reclined on a bean bag (categorized as prone supported positions) (S1 File). The infants were placed in each position by their parent for approximately 3 minutes each. The entire session was video recorded and the position of the baby was noted for each second. Parents also completed a 25-item questionnaire to understand their perceptions of the practicality of their infant wearing each device at home (S2 File).

### Devices

The ActiGraph and GENEActiv devices are tri-axial accelerometry-based activity monitors (ActiGraph LLC, Pensacola, FL, USA; Activinsights Ltd, Cambridgeshire, UK). They were initialized with a sampling frequency of 30 Hz and set with on and off times to record the data. The ActiGraph accelerometers (hip and ankle) were orientated (baby supine) with the x-axis pointing vertically forwards (front to back), y-axis horizontally (head to feet) and z-axis horizontally (hip to hip). The MonBaby is composed of a circuit board, which incorporates a precision 14-bit, tri-axial accelerometer sensor chip that communicates with the BLE microprocessor chip (MonDevices Inc, New York, NY, USA). MonBaby has a sample frequency of 6.25 Hz and begins to record immediately once it is configured (i.e. put flat on a table so the device is aware when it is face up or upside-down). MonBaby is orientated (baby supine) with the x-axis pointing horizontally (side to side), y-axis vertically (head to feet) and the z-axis facing forwards (front to back).

After each session, the individual GENEActiv data were uploaded and the raw .bin files converted to 1s epoch .csv files using GENEActiv PC software (version 2.2). The 1s epoch files from the GENEActiv device were imported into custom-built spreadsheets in Excel that computed the most likely position (classified as prone, non-prone or prone supported) using an algorithm developed by Activinsights Ltd. This algorithm was formed by classifying each position from a scatter plot with rotation (360 degree angle) on the x-axis and elevation (up/down angle) on the y-axis (S3 File). The GENEActiv accelerometer was oriented (baby supine) with the x-axis horizontally to the right (side to side), y-axis pointing towards the feet (head to feet), and z-axis facing forward (front to back). In addition, as the movement was low, the signal from the accelerometer is dominated by the Earth’s gravitational field, as such, the angles can be calculated without a gyroscope [26].

Similarly, the ActiGraph data were uploaded and the raw .GTx file converted to 1s epoch .csv file using ActiGraph PC software (version 6.12.1). A custom-built Excel macro designed by ActiGraph then opened the .csv file and position (classified as prone, non-prone and prone supported for ActiGraph Hip; prone and non-prone for ActiGraph Ankle) was determined. This was achieved by using specified x and y axis cut-points for ActiGraph Hip (x axis >0.7g and y axis >-0.1g for prone on floor; x axis >0.7g and y axis <-0.1g for prone supported) and x and z axis cut-points for ActiGraph Ankle (x axis >0.35g and z axis >-0.45g for prone). Correction for gravity is done automatically upon downloading the data into the Macro for analysis, as such, a Gyroscope was not required.

For MonBaby, a custom-built dashboard was developed by MonDevices Inc. A Universal Unique Identifier (UUID) was used by the dashboard to retrieve the data collected (timestamp, x,y and z axis) by the MonBaby device from each testing session. Data were then downloaded from the dashboard into a .csv file and then converted into an Excel file. For the non-prone positions, a formula was copied into each row to determine the 360 degree angle calculated from the x, y and z axis [= ACOS(E3/SQRT(C2*C2+D2*D2+E2*E2))*180/PI() with the number representing the row on the Excel spreadsheet and C being the x axis, D the y axis, E the z axis]. All 360-degree angles that were calculated as less than 134 degrees were classified as non-prone. Prone on floor and prone supported positions were determined by using a z axis cut-point of <-0.10g. Downloading data from the MonBaby device required a manual entry of the time period in question (yyyy/mm/dd hh:mm:ss to yyyy/mm/dd hh:mm:ss). When downloaded, the data is presented as 5 to 7 epochs per second (5–7 rows) rather than 1s per row on the excel spreadsheet. As such, the percentage accuracy of the section downloaded was determined rather than second by second analysis.

### Direct observation

The criterion measure was direct observation captured on a video recording of the whole session. Infants were recorded completing each position in addition to transitioning between each position. A single observer coded each second of the video-recording using the definitions outlined in supporting information 1 (S1 File). One randomly selected video was analyzed by four observers to test inter-observer reliability. Each second in the video was analyzed by each observer. Of the 4251 seconds analyzed, 97.5% were recorded to be the same position for all 4 raters.

### Statistical analyses

The position codes from direct observation served as the criterion measure for all four devices. The percentage accuracy of the devices to measure prone on floor, non-prone and prone supported positions for the total time of the positioning protocol were analyzed. For ActiGraph (hip and ankle) and GENEActiv, this was done by calculating the number of seconds recorded by each device in the 3 categories (prone on floor, non-prone and prone supported) and comparing to the seconds recorded by direct observation in the same three categories. Time spent (duration in seconds) in each category was evaluated for each device against direct observation. For MonBaby, only the percentage accuracy of the section downloaded and comparing to the same section of time from direct observation was calculated. This was then converted into seconds to enable a comparison between the devices. Bland-Altman plots were used to examine systematic bias in estimates of time spent in each category from each device [27].

## Results

Participant information is shown in Table 1. All participants (N = 32) completed the positioning protocol and had data available from each device to review for each category of position except for MonBaby where there was no data downloaded from three prone on floor and three prone supported categories due to connectivity issues. The complete duration of all 32 videos was 98150 seconds. Infants were coded as: off screen (for example, mother took baby off screen to breast feed, mother in front of infant on video thus blocking the view) for 8% of this time; were coded as invalid position (for example, when the infants were not in one of the 12 defined positions such as picking up off the floor, putting into the car seat/pram, supported standing on the lap of the parent, infant being held whilst parent is moving into and out of the beanbag) for 18% of this time; and coded as invalid device (for example, if the device required re-positioning on the infant) for <1% of the time. The total time able to be classified as one of the 12 positions (excluding when the infant was coded as off screen, invalid position or invalid device) was 72050 seconds (11749 seconds in prone positions; 49123 seconds in non prone positions and 11178 seconds in prone supported positions).

**Table 1:** Participant characteristics.

Infants (N = 32)	
Age (weeks)	15.20 ± 6.39, *(4*.*71 to 24*.*86)*
Boys (n) Girls (n)	19 (59.4%) 13 (40.6%)
Recumbent length (cm)	61.01 ± 3.49, *(52*.*0 to 68*.*0)*
Weight (kg)	6.28 ± 1.25, *(4*.*34 to 9*.*22kg)*
Complex medical conditions	0

Participant characteristics presented as mean ± SD and range. The distribution of the sample is presented in numbers (n) and percentages (%).

**Fig 2:**
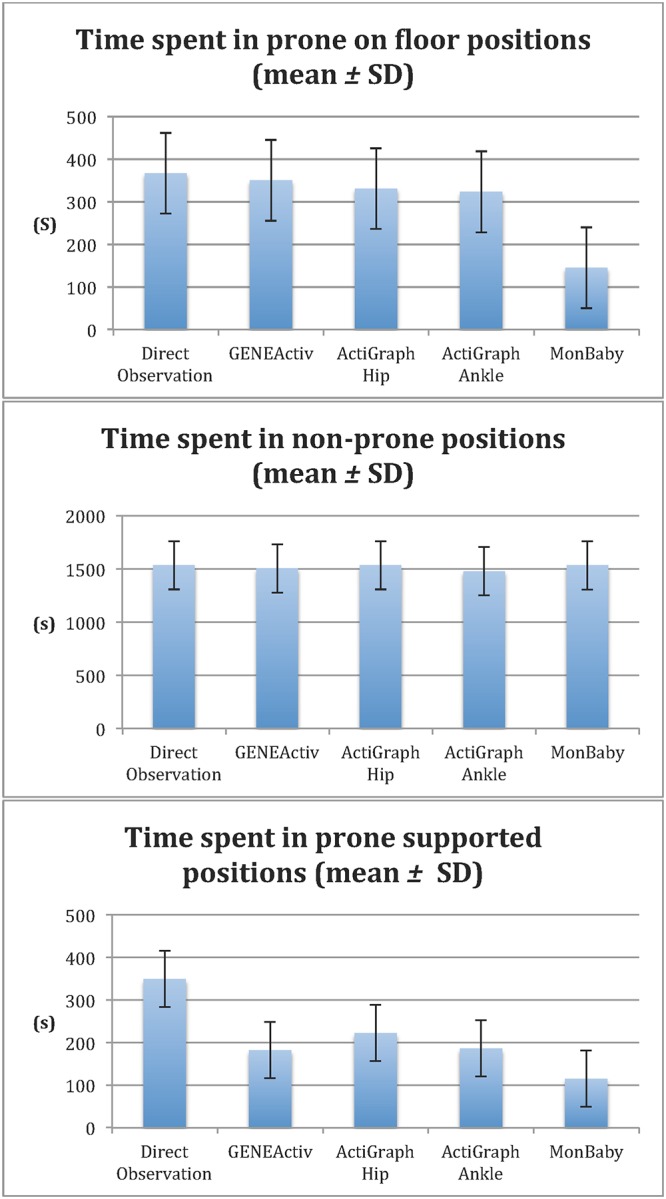
Time spent in prone on floor, non-prone and prone supported positions.

For the total time, the most accurate algorithms or cut-points were from GENEActiv for the prone on floor positions (95.4%), and both the ActiGraph Hip (99.9%) and MonBaby (99.9%) for the non-prone positions. MonBaby was the most accurate for the prone supported positions (66.1%) (Fig 2 and Table 2), however, none of the algorithms had acceptable levels of accuracy for this position.

**Table 2:** Seconds recorded by the criterion measure and the four devices for the different categories of positions.

	Direct observation (criterion measure)			GENEActiv			ActiGraph Hip			ActiGraph Ankle			^#^MonBaby		
Positions	Prone on floor	Non prone	Prone supported	Prone on floor	Non prone	Prone supported	Prone on floor	Non prone	Prone supported	Prone on floor	Non prone	Prone supported	Prone on floor	Non prone	Prone supported
Duration (s)	11749	49123	11178	11209	48124	5836	10576	49118	7105	10333	47313	5959^	9302	49071	7387^
Accuracy (%)	100	100	100	95.4	98	52.2	90	99.9	63.6	87.9	96.3	53.3	79.2	99.9	66.1^
Average (s)	367	1535	349	350	1504	182	331	1535	222	323	1479	186	145	1533	115
SD (s)	98	227	66	105	232	100	112	227	108	103	245	120	95	227	84

Data presented as duration (s), accuracy (% accuracy compared to direct observation), average (s) ± SD (s) for all 32 babies. GENEActiv and ActiGraph Hip and Ankle data presented as seconds measured by each device respectively.

^#^MonBaby data presented as seconds which has been estimated from percentage accuracy of the section downloaded from the MonBaby device for each time period.

^MonBaby and ActiGraph ankle were unable to differentiate between prone supported and prone positions. As such, for these devices, held in prone and prone on the parent’s chest positions (prone supported) are recorded as prone.

The GENEActiv appeared to have some difficulty classifying right sidelying (Table 3) with approximately 14% of right sidelying positions recorded as prone. Due to connectivity issues, MonBaby had difficulty recording prone on floor and prone supported positions. The ActiGraph Ankle had difficulty with classifying being held upright with approximately 24% of upright positions being classified as prone (Table 3).

**Table 3:** Total seconds prone recorded by direct observation, GENEActiv, ActiGraph Hip and ActiGraph Ankle for each position (N = 32).

	Direct observation (s) *(criterion measure)*	GENEActiv (s)	ActiGraph Hip (s)	ActiGraph Ankle (s)
Supine	0	0	1	19
Prone attempt 1	5763	5668	5295	5126
Left sidelying	0	267	0	388
Right sidelying	0	732	4	8
Cradle hold	0	0	0	9
Held in prone	5546	2932	2899	2256
Prone on parent’s chest	5632	2904	4206	3703
Car seat	0	0	0	0
Held upright	0	0	0	1325
Supported sitting	0	0	0	61
Prone attempt 2	5986	5541	5281	5207
Pram	0	0	0	0

Data presented in total seconds; N = 32; Held in prone and prone on parent’s chest positions are recorded as prone supported for GENEActiv and ActiGraph Hip and prone for ActiGraph Ankle.

**Fig 3:**
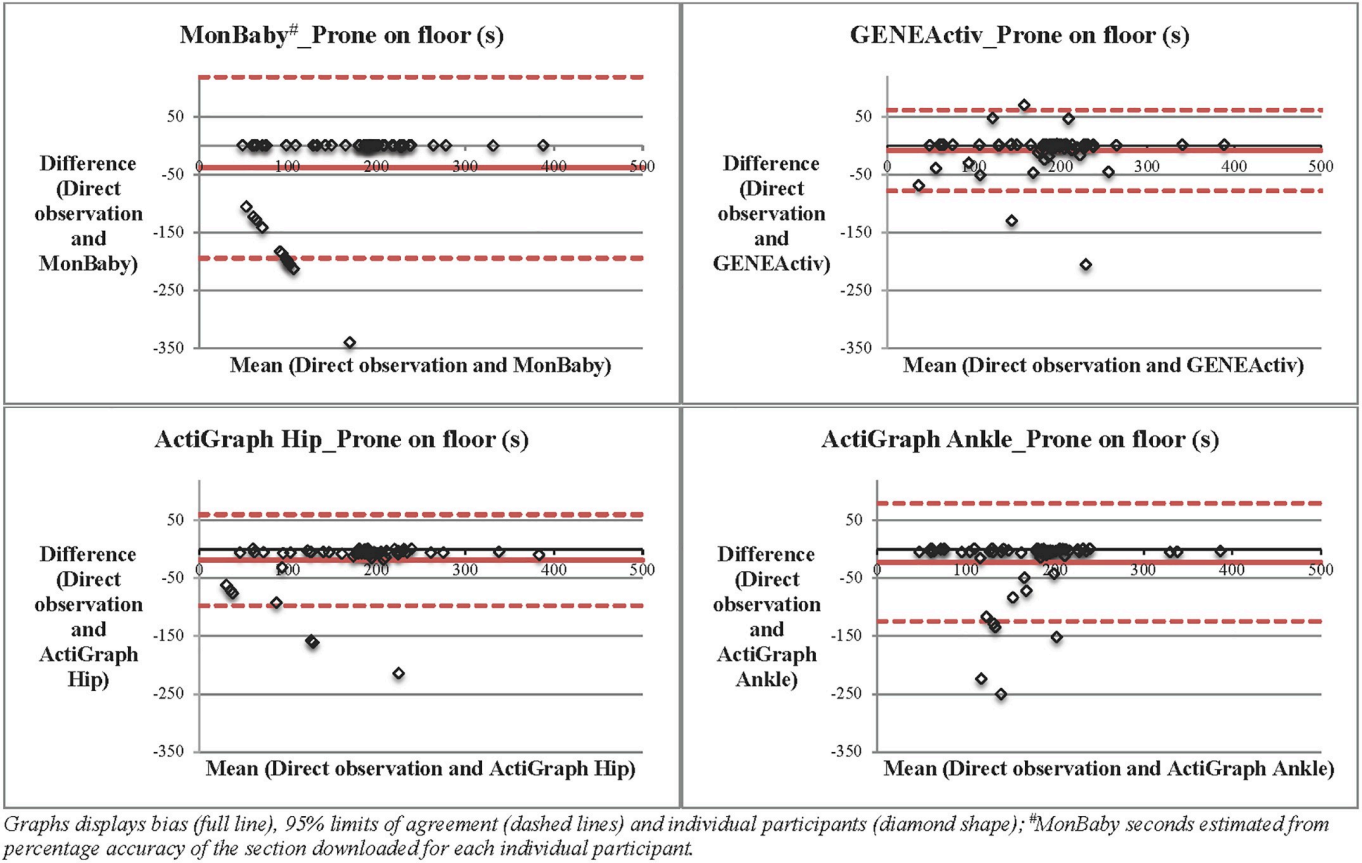
Bland-Altman plots of MonBaby, GENEActiv, ActiGraph Hip and ActiGraph Ankle for prone on floor positions. Graphs displays bias (full line), 95% limits of agreement (dashed lines) and individual participants (diamond shape); ^#^MonBaby seconds estimated from percentage accuracy of the section downloaded for each individual participant.

Bland–Altman procedures (Fig 3) demonstrated underestimation for prone on floor, prone supported and non-prone position categories. For prone on floor positions, GENEActiv had the smallest mean difference (-8.4s) and limits of agreement than ActiGraph Hip (-18.3s), ActiGraph Ankle (-22.1s) and MonBaby (-38.0s). Bland-Altman procedures for prone supported categories demonstrated the largest difference and limits of agreement for all devices. For these prone supported positions, MonBaby had the smallest difference (-113.0s), compared with ActiGraph Hip (-127.3s), ActiGraph Ankle (-163.1s) and GENEActiv (-166.9s). Bland-Altman procedures for non-prone categories demonstrated the smallest difference and limits of agreement for all devices. For these non-prone positions, ActiGraph Hip had the smallest difference (-0.2s) compared with MonBaby (-2.0s), GENEActiv (-31.2s) and ActiGraph Ankle (-56.6s). Table 4 shows the limits of agreement for each device’s algorithm or cut-point when compared to direct observation. Additional Bland-Altman plots for prone supported and non-prone positions can be found in supporting information (S4 File).

**Table 4:** Values (LoA^) when compared to direct observation from Bland-Altman procedures.

Positions	Values (LoA^) as measured by each algorithm (s)			
	GENEActiv	ActiGraph Hip	ActiGraph Ankle	MonBaby
Prone on floor	-8.4(-78.2 to 61.3)	-18.3(-97.0 to 60.3)	-22.1(-124.0 to 79.7)	-38(-194.5 to 118.5)
Non-prone	-31.2(-154.9 to 92.4)	-0.2(-1.2 to 0.9)	-56.6(-209.5 to 96.3)	2.0(-14.0 to 10.8)
Prone supported	-166.9(-390.7 to 56.8)	-127.3(-324.7 to 70.2)	-163.1(-431.7 to 105.6)	-113(-355.6 to 129.6)

^limits of agreement

Practicality questionnaire results are shown in Table 5. Most parents thought all devices did not interfere with infant positioning (90.6%), the infant’s ability to move (91.4%), were not uncomfortable (93.8%), required little effort to put the device on and off (82.8%), were not difficult to attach (97.7%) and could be worn for more than 3 days (85.2%). Out of the four devices, most parents (78.1%) rated the MonBaby device as the most preferable device for their baby to wear. Approximately 38% and 34% of parents rated the ActiGraph Ankle and GENEActiv devices respectively as their second preference with 59% of parents rating ActiGraph Hip as their fourth preference.

**Table 5:** Practicality questionnaire—Number of parents and percentage of agreement (N = 32).

The device:	MonBaby	GENEActiv	ActiGraph Hip	ActiGraph Ankle
Did not interfere with the positions my baby was placed in today	32 (100%)	29 (90.6%)	25 (78.1%)	30 (93.8%)
Did not interfere with my baby’s ability to move around freely	32 (100%)	30 (93.8%)	26 (81.3%)	29 (90.6%)
Was not uncomfortable for my baby to wear (including attaching and removing devices)	32 (100%)	30 (93.8%)	28 (87.5%)	30 (93.8%)
Did not require a lot of input to ensure the device was kept on correctly	26 (81.3)	28 (87.5%)	26 (81.3%)	27 (84.4%)
Was able to be attached by myself	31 (96.9%)	32 (100%)	31 (96.9%)	31 (96.9%)
Could be tolerated by my baby to wear (during the daytime) for at least 3 days	31 (96.9%)	26 (81.3%)	30 (93.8%)	28 (87.5%)

Practicality questionnaire results presented as number (n) and percentages (%)

Internal consistency as measured by Cronbach’s Alpha, α = 0.91

## Discussion

This study investigated the accuracy of the manufacturer’s (MonDevices Inc, ActiGraph LLC and Activinsights Ltd) algorithm or cut-points for predicting posture. The GENEActiv device was the most accurate to determine prone on floor positions, followed by ActiGraph Hip. All devices were able to accurately determine non-prone positions but not prone supported positions.

The evaluated algorithms for GENEActiv and ActiGraph (hip) were able to accurately record and calculate when an infant was positioned prone on the floor (>90% accuracy). All algorithms were able to accurately record and calculate when an infant is placed in a non-prone position (such as supine on the floor, car seat, pram, and supported sitting) with greater than 95% accuracy. To our knowledge, no previous studies have evaluated the validity of an accelerometer or objective measurement tool to assess infant positioning practices and tummy time. Although MonBaby was most preferred by parents, improvements in connectivity would be required before recommending MonBaby for use in further studies. For prone position detection, the cut-point for MonBaby was 100% accurate at identifying tummy time when there was no connectivity issues, and 79% accurate when taking into account data loss. Data connectivity issues resulted from mothers or babies blocking the signal of the device with their bodies. For future studies investigating objective measurement tools for tummy time, MonBaby would require additional testing. Additionally, it is important to note that MonBaby has been designed as a “Smart Breathing Movement Monitor for Babies” and not to measure prone positioning (tummy time). As such, the results from this study do not necessarily reflect the purpose for which it was originally designed.

The algorithms or cut-points had difficulty correctly classifying prone supported (GENEActiv, ActiGraph Hip) or prone (ActiGraph Ankle, MonBaby). Accuracy was less than 70% for all devices. Further analysis and adjustments to the cut-points and algorithms could be made to increase this accuracy level to a more acceptable standard. However, for future studies where the outcome is to objectively measure the time an infant spends in tummy time when it is defined as awake and supervised prone positioning on the floor, GENEActiv and ActiGraph devices would be the most suitable to use.

This study also investigated the practicality of using devices on infants as an objective measure of tummy time. All devices were found to be practical and feasible to use by the parents. This finding is similar to previous studies that have investigated the use of accelerometers to measure physical activity levels in toddlers [28]. Most studies place the accelerometer on the child’s ankle [28] or hip [23, 29]. However, in this study, as the participants were infants, the positioning of the devices on the hip and ankle with a strap were not as amenable to parents as MonBaby’s ‘smart button’ which attaches to the infant’s clothing in the middle of the infant’s chest. Most parents (78.1%) preferred the MonBaby device as it was the least intrusive (was thinner than the other devices), able to be left on for nappy changes, the least likely to move out of position and able to be positioned on the infant’s clothes at the level of their chest without a strap. For all devices, a singlet plus a full ‘wondersuit’ (arms and legs included) or ‘onesie’ are recommended to ensure correct device placement and to avoid redness on the chest, waist and ankle (S5 File).

Extrapolations from the findings of this study need to take into consideration that the positioning protocol was laboratory-based which may not reflect the infant’s real world positions. To account for this, parents were asked to place the infant in the different positions in an attempt to keep positioning as realistic as possible. In addition, an analysis of the wear and non-wear times is required before using one of these devices as an objective tummy time measure without requiring the parents to log or record these times. In addition, the placement of GENEActiv and ActiGraph around the infant’s waist was not randomized. This could account for GENEActiv recording right sidelying as prone for approximately 14% of the total time. This may be due to its position being closer than ActiGraph to the midline of the infant’s body (Fig 1). It is expected that if GENEActiv was relocated to the tested location of the ActiGraph Hip, this percentage would be reduced.

Further research could include an investigation of the effect of infants being placed on the floor versus being held in prone [30] and the infant’s ability in tummy time (rather than only duration in tummy time) to infant health outcomes [31]. In addition, further investigation into the application of the devices to assess non-prone positions particularly the amount of time spent restrained would also be valuable, as this has been identified as a specific recommendation in the 24-hour Movement Guidelines. Rigorous assessment of potential variables and objective outcome measures could be an effective way of ensuring a more consistent method of assessing the impact of infant positioning in relation to the 24-hour movement guidelines.

### Conclusion

The algorithms evaluated in this study for GENEActiv and ActiGraph (hip) were of acceptable accuracy to objectively measure the amount of time an infant spends in tummy time (when it is defined as awake and supervised prone positioning on the floor). MonBaby was most preferred by parents due to its positioning on the chest and unobtrusive design. Improvements would be required before recommending MonBaby for use in future studies. Future research could be conducted to identify wear and non-wear times as well as identifying individual non-prone positions (for example, finding an algorithm to separate out being reclined in a pram, supported sitting and supine). Accurate measurement of infant positioning practices will be important to assist in the observance of 24-hour movement guidelines in the early years.

#### Practical implications

- The algorithms evaluated in this study for GENEActiv and ActiGraph (hip) are of suitable accuracy to objectively measure tummy time (awake and supervised prone positioning on the floor) in infants
- MonBaby, GENEActiv and ActiGraph devices are practical and feasible to be used by parents
- Most parents preferred the MonBaby “smart button”, chest position and unobtrusive design
- Potential over reporting of tummy time could occur from right sidelying (GENEActiv) and being held upright (ActiGraph Ankle)
- Objective measures of tummy time and other infant positions may assist in the observance of 24-hour movement guidelines in the early years.

## Supporting information

S1 File Definitions of positions used to code video analysis. (PDF)

S2 File Practicality questionnaire. (PDF)

S3 File Algorithm for GENEActiv device to classify prone, non-prone and prone supported positions. (PDF)

S4 File Bland-Altman plots for non-prone and prone supported positions. (PDF)

S5 File Device parameters. (PDF)
