# Supplementary material for: Objective measurement of tummy time in infants (0-6 months): A validation study
Source: PLoS One. 2019 Feb 27;14(2):e0210977. doi: 10.1371/journal.pone.0210977 (PMC6392225; doi:10.1371/journal.pone.0210977)
Supplement: S1 File — (PDF) [file pone.0210977.s001.pdf]

Supporting information 1. Definitions of positions used to code video analysis

Category	Position	Definition	Picture
Prone positions	Prone attempt 1 and 2	Baby lying on the floor on their tummy. Both hips touching ground. Ok to push up with their arms and lift head off the ground or keep head on the ground. Must be awake and supervised for safety.	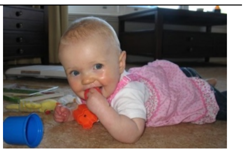 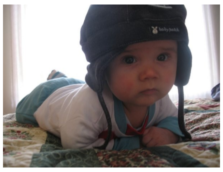
Non prone positions	Supine	Baby lying on the floor on their back. Both shoulders and hips touching ground. Able to kick freely but not roll over or onto their side.	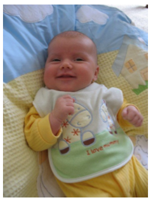
	Left sidelying	Baby on floor with their left shoulder and hip touching the ground. Reclined leaning on rolled up towel. Not to either roll forward or roll back onto floor but stay as midline as possible.	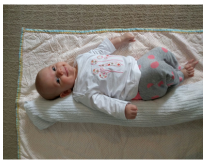
	Right sidelyling	Baby on floor with their right shoulder and hip touching the ground. Reclined leaning on rolled up towel. Not to either roll forward or roll back onto floor but stay as midline as possible.	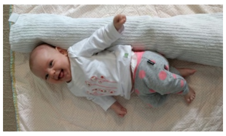
	Reclined in car seat	Baby reclined in a car seat. Seat belt fastened.	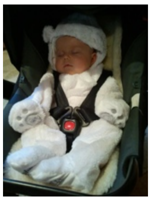
	Upright	Baby held upright over the parent's shoulder.	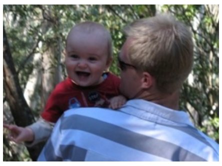
	Reclined in pram	Baby reclined in a pram. Seatbelt on.	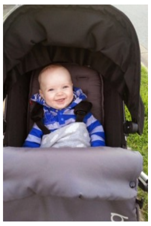

	Supported sitting	Baby held in supported sitting on the parent's lap who is sitting in a chair.	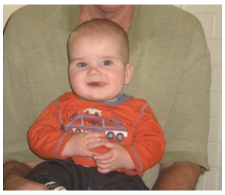
	Cradle hold	Baby held in crook of parent's arm or feeding baby (bottle or breast).	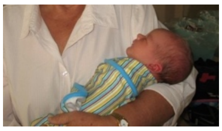
Prone supported positions	Prone on parent's chest	Parent reclined on a beanbag. Parent's head, shoulders and bottom are touching the beanbag. Baby on tummy lying on parent's chest with their head near the parent's chin or shoulder. Baby placed straight.	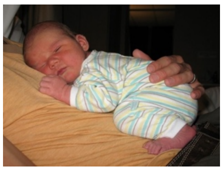
	Being held in prone	Baby held prone whilst parent is standing or sitting. Parent can sway hips from side to side or gently rock baby if needed.	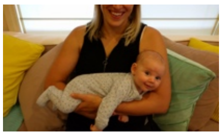
Other positions	Off screen	Baby is currently not on the video screen. For example, parent is in front of the baby or parent took baby off screen to feed/change nappy/settle.	
	Invalid position	Baby is in a position other than the ones stated above. For example, supported standing, getting in and out of the car seat/pram, being held whilst the parent is getting into the beanbag, picking up off the floor.	
	Invalid device	Device is not in the correct position. For example, strap moved around, device flipped upside-down.	
