# Supplementary material for: Objective measurement of tummy time in infants (0-6 months): A validation study
Source: PLoS One. 2019 Feb 27;14(2):e0210977. doi: 10.1371/journal.pone.0210977 (PMC6392225; doi:10.1371/journal.pone.0210977)
Supplement: S2 File — (PDF) [file pone.0210977.s002.pdf]

Practicality Questionnaire

Instructions: Please mark your response to the statement in the corresponding box provided, example: ☑

**MonBaby on CHEST:**	Strongly disagree	Disagree	Neither agree or disagree	Agree	Strongly agree
The MonBaby interfered with the positions my baby was placed in today	☐	☐	☐	☐	☐
The MonBaby interfered with my baby's ability to move around freely	☐	☐	☐	☐	☐
The MonBaby was uncomfortable for my baby to wear (including attaching and removing devices)	☐	☐	☐	☐	☐
A lot of input was required to ensure the MonBaby was kept on correctly	☐	☐	☐	☐	☐
I would not be able to attach the MonBaby by myself	☐	☐	☐	☐	☐
My baby would not be able to tolerate wearing the MonBaby (during the daytime) for at least 3 days	☐	☐	☐	☐	☐

**ActiGraph on HIP:**	Strongly disagree	Disagree	Neither agree or disagree	Agree	Strongly agree
The ActiGraph interfered with the positions my baby was placed in today	☐	☐	☐	☐	☐
The ActiGraph interfered with my baby's ability to move around freely	☐	☐	☐	☐	☐
The ActiGraph was uncomfortable for my baby to wear (including attaching and removing devices)	☐	☐	☐	☐	☐
A lot of input was required to ensure the ActiGraph was kept on correctly	☐	☐	☐	☐	☐
I would not be able to attach the ActiGraph by myself	☐	☐	☐	☐	☐

**My baby would not be able to tolerate wearing the ActiGraph (during the daytime) for at least 3 days**	☐	☐	☐	☐	☐

**ActiGraph on ANKLE:**	**Strongly disagree**	**Disagree**	**Neither agree or disagree**	**Agree**	**Strongly agree**
**The ActiGraph interfered with the positions my baby was placed in today**	☐	☐	☐	☐	☐
**The ActiGraph interfered with my baby's ability to move around freely**	☐	☐	☐	☐	☐
**The ActiGraph was uncomfortable for my baby to wear (including attaching and removing devices)**	☐	☐	☐	☐	☐
**A lot of input was required to ensure the ActiGraph was kept on correctly**	☐	☐	☐	☐	☐
**I would not be able to attach the ActiGraph by myself**	☐	☐	☐	☐	☐
**My baby would not be able to tolerate wearing the ActiGraph (during the daytime) for at least 3 days**	☐	☐	☐	☐	☐

**GENEActiv on HIP:**	**Strongly disagree**	**Disagree**	**Neither agree or disagree**	**Agree**	**Strongly agree**
**The GENEActiv interfered with the positions my baby was placed in today**	☐	☐	☐	☐	☐
**The GENEActiv interfered with my baby's ability to move around freely**	☐	☐	☐	☐	☐
**The GENEActiv was uncomfortable for my baby to wear (including attaching and removing devices)**	☐	☐	☐	☐	☐
**A lot of input was required to ensure the GENEActiv was kept on correctly**	☐	☐	☐	☐	☐
**I would not be able to attach the GENEActiv by myself**	☐	☐	☐	☐	☐

**My baby would not be able to tolerate wearing the GENEActiv (during the daytime) for at least 3 days**	☐	☐	☐	☐	☐

Please rate from 1 to 4 (with 1 being the most preferable) which monitor you would prefer your baby to wear:

☐	☐	☐	☐
MonBaby on chest	ActiGraph on Hip	ActiGraph on Ankle	GENEActiv on hip
