# Supplementary material for: Objective measurement of tummy time in infants (0-6 months): A validation study
Source: PLoS One. 2019 Feb 27;14(2):e0210977. doi: 10.1371/journal.pone.0210977 (PMC6392225; doi:10.1371/journal.pone.0210977)
Supplement: S3 File — (PDF) [file pone.0210977.s003.pdf]

S3 File. Algorithm for GENEActiv device to classify prone, non-prone and prone supported positions.

The algorithm calculated:

- 360 Angle [SIGN(B101)*ACOS(-D101/SQRT(B101^2+D101^2))*180/PI()+180][1];
- up/down angle [ASIN(C101/(B101^2+C101^2+D101^2)^0.5)*180/3.14] [2];
- body rotation [IF(AND(N101>S5, N101<(S5+180)),"supine-recline","prone-sit"][3];
- prone-sit class [IF(AND(Q101="prone-sit",O101>0),"prone",IF(AND(Q101="prone-sit",O101>-23),"prone supported",IF(AND(Q101="prone-sit",O101>-63),"upright",IF(Q101="prone-sit","sitting",""))))][4];
- Supine-recline class [IF(AND(Q101="supine-recline",O101>15),"upsidedown",IF(AND(Q101="supine-recline",O101<-36),"reclined",IF(AND(Q101="supine-recline",N101<(S5+69)),"left side",IF(AND(Q101="supine-recline",N101>(S5+101)),"right side",IF(Q101="supine-recline","supine","")))))][5]; and then
- Overall class [CONCATENATE(R101,S101)] [6].
- Final outcome of infant position was taken from the overall class. (where B is x-axis, C is y-axis, D is z-axis, N is 360 degree angle, O is up/down angle, P is observed position, Q is body rotation, R is prone-sit class, S is supine-recline class, T is overall class and S5 is a prone to supine angle of 140 degrees).
