# Supplementary material for: Objective measurement of tummy time in infants (0-6 months): A validation study
Source: PLoS One. 2019 Feb 27;14(2):e0210977. doi: 10.1371/journal.pone.0210977 (PMC6392225; doi:10.1371/journal.pone.0210977)
Supplement: S4 File — (PDF) [file pone.0210977.s004.pdf]

S4_File. Bland Altman plots for non-prone and prone supported positions

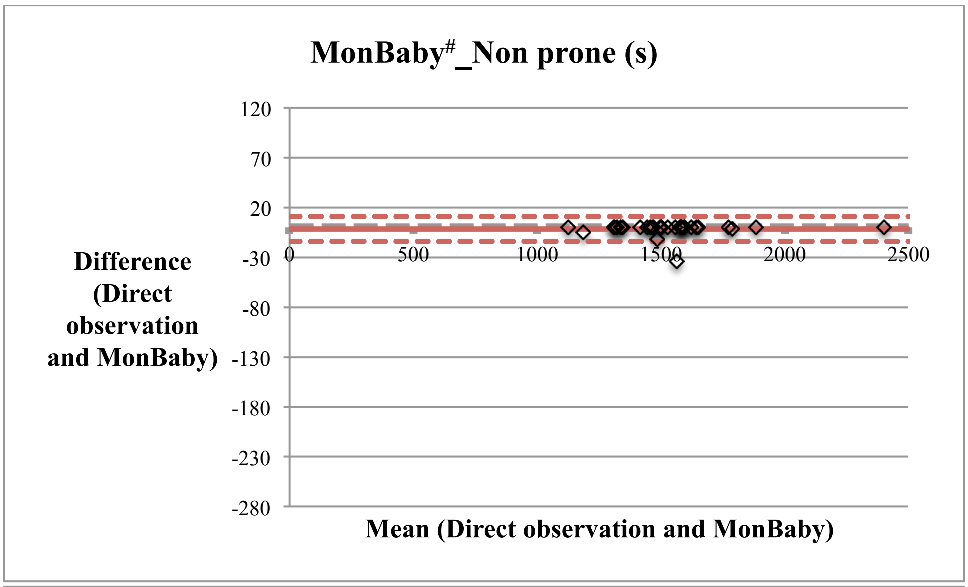

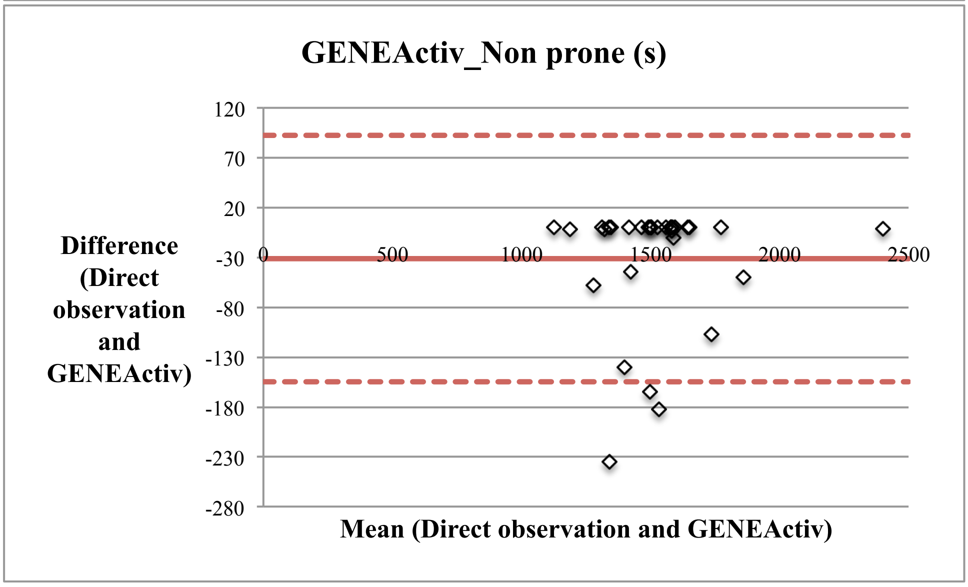

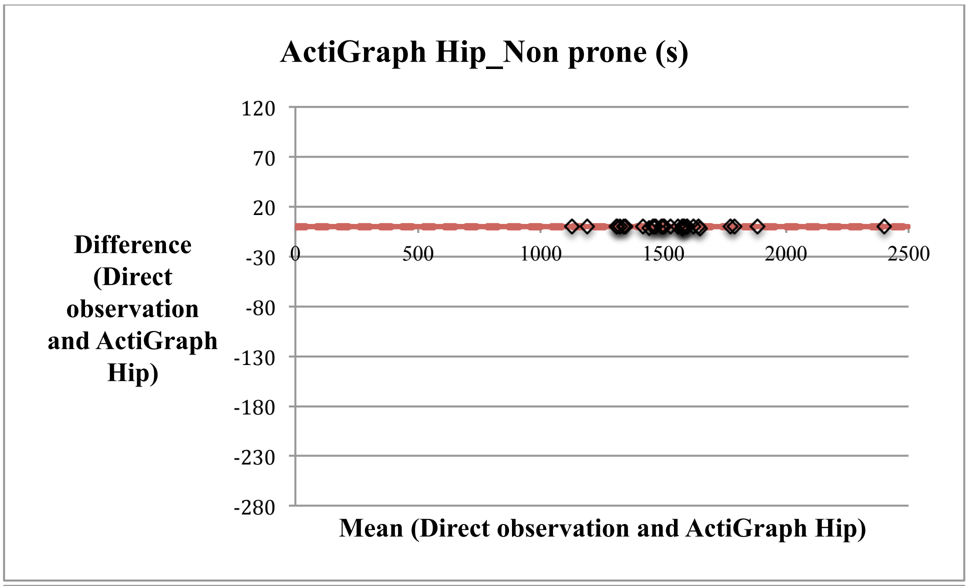
ActiGraph Hip_Non prone (s)
120
70
20
-30
-80
-130
-180
-230
-280
0
500
1000
1500
2000
2500
Difference (Direct observation and ActiGraph Hip)
Mean (Direct observation and ActiGraph Hip)

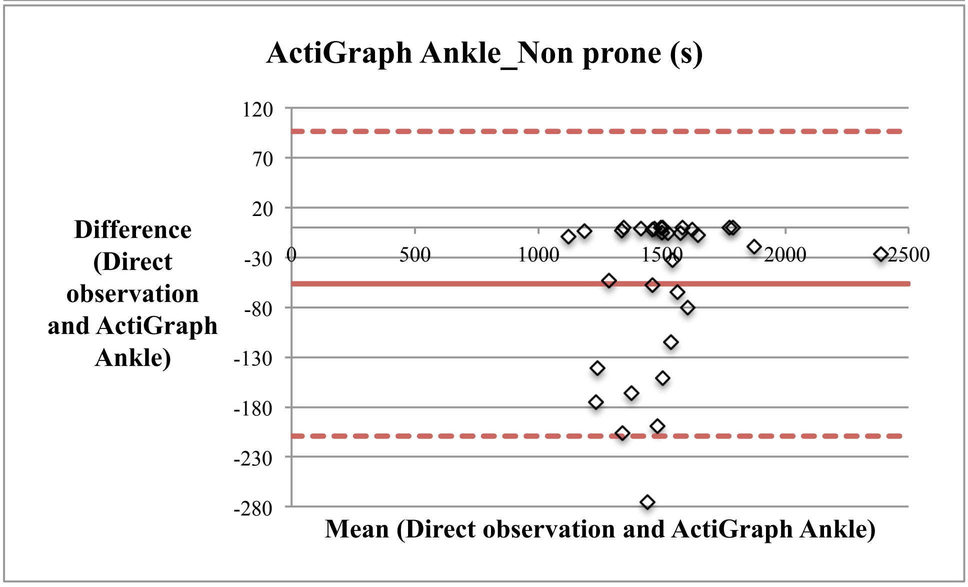
ActiGraph Ankle_Non prone (s)
120
70
20
-30
-80
-130
-180
-230
-280
0
500
1000
1500
2000
2500
Difference (Direct observation and ActiGraph Ankle)
Mean (Direct observation and ActiGraph Ankle)

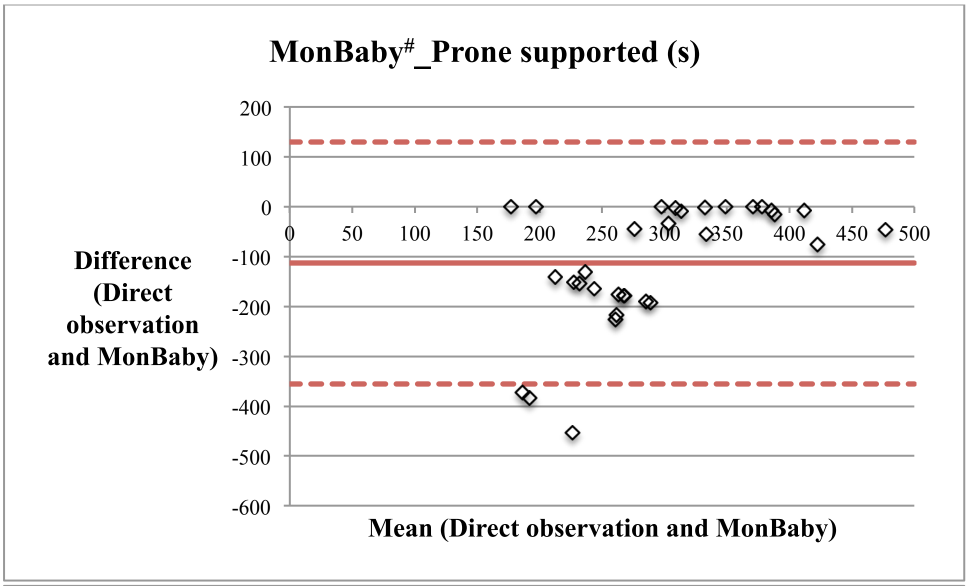
MonBaby#_Prone supported (s)
Difference (Direct observation and MonBaby)
200
100
0
-100
-200
-300
-400
-500
-600
0
50
100
150
200
250
300
350
400
450
500
Mean (Direct observation and MonBaby)

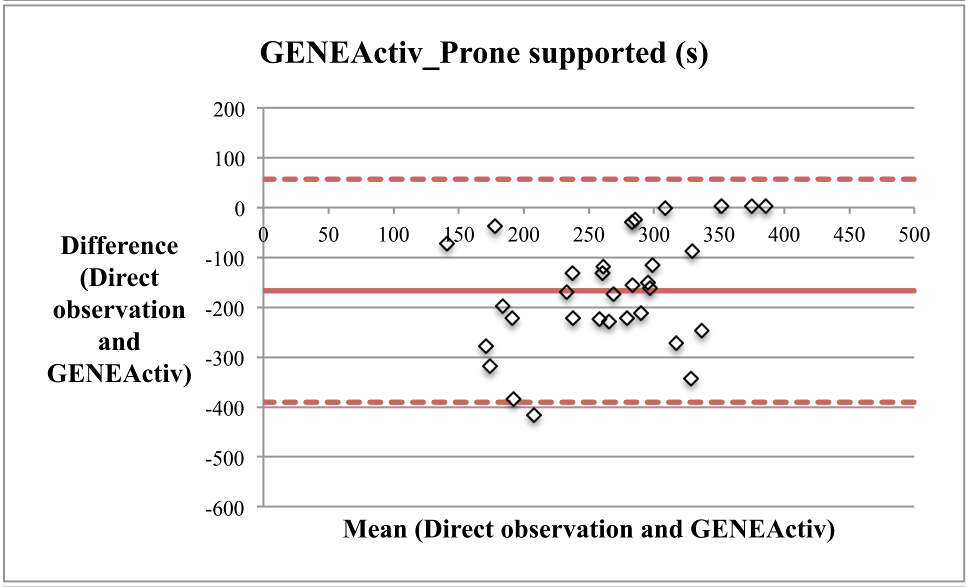
GENEActiv_Prone supported (s)
Difference (Direct observation and GENEActiv)
200
100
0
-100
-200
-300
-400
-500
-600
0
50
100
150
200
250
300
350
400
450
500
Mean (Direct observation and GENEActiv)

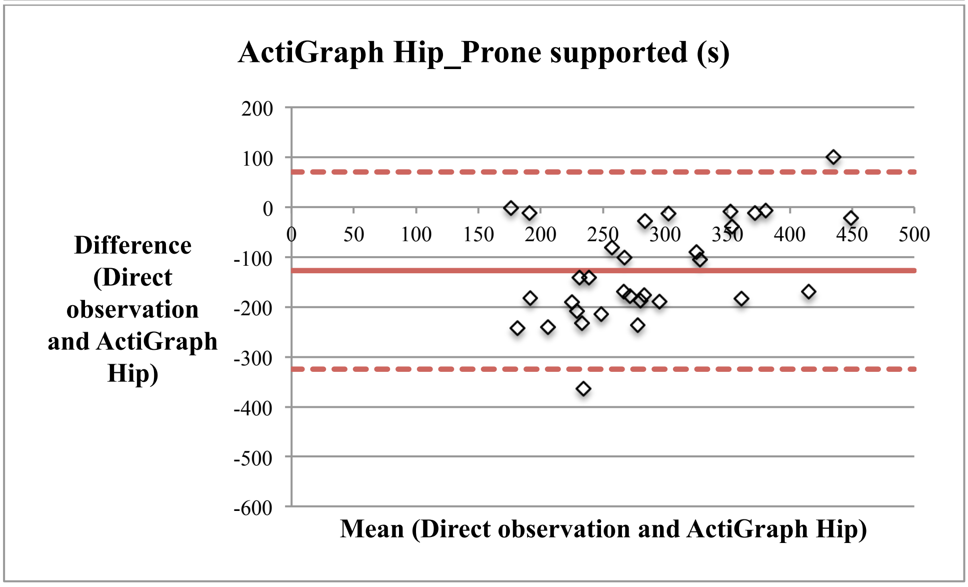
ActiGraph Hip_Prone supported (s)
Difference (Direct observation and ActiGraph Hip)
200
100
0
-100
-200
-300
-400
-500
-600
0
50
100
150
200
250
300
350
400
450
500
Mean (Direct observation and ActiGraph Hip)

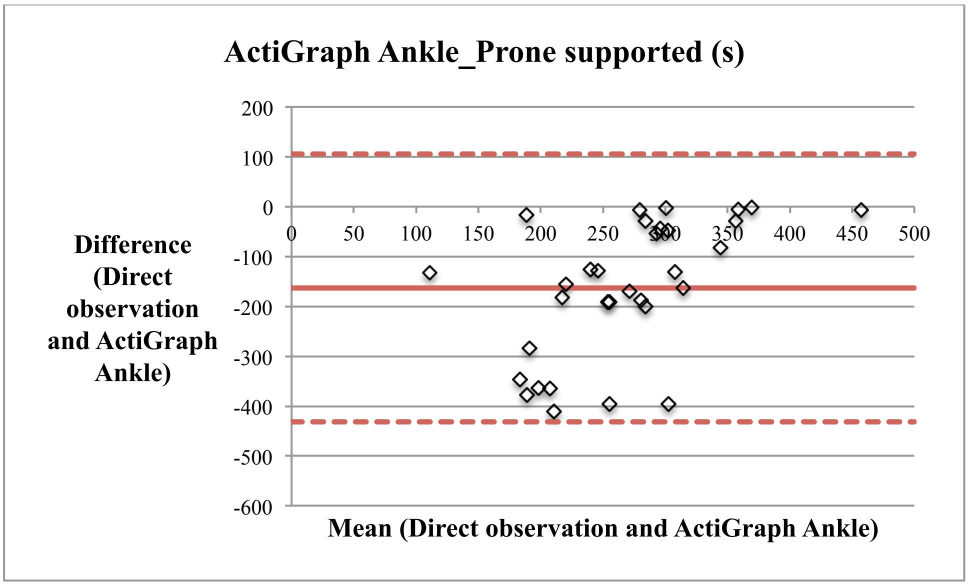

Graphs displays bias (full line), 95% limits of agreement (dashed lines) and individual participants (diamond shape); #MonBaby seconds estimated from percentage accuracy of the section downloaded for each individual participant
