# Supplementary material for: Objective measurement of tummy time in infants (0-6 months): A validation study
Source: PLoS One. 2019 Feb 27;14(2):e0210977. doi: 10.1371/journal.pone.0210977 (PMC6392225; doi:10.1371/journal.pone.0210977)
Supplement: S5 File — (PDF) [file pone.0210977.s005.pdf]

S5 File. Device parameters#

	MonBaby	GENEActiv	ActiGraph Hip	ActiGraph Ankle
Size	37mm x 37mm x 8mm	43mm x 40mm x 13mm	38mm x 40mm x 15mm (includes clip)	38mm x 40mm x 15mm (includes clip)
Weight	10.3g including battery and smart button clasp	16g without strap	29.2g including clip but without strap	29.2g including clip but without strap
Battery life	Dependent upon environment for signal strength	21 days when recording at 30Hz*	14 days when recording at 30Hz+	14 days when recording at 30Hz+
Software	Free to download app onto iPhone or Android phone after purchase of device	Free to download	Requires licence	Requires licence
Cost (AUS dollars)	$101.34 + shipping	$388 + shipping	$275 + accessories and single licence ($1695)	$275 + accessories and single licence ($1695)
Infant clothing required	Smart button clasp onto clothes on infant's chest (included). Onesie and singlet underneath	Onesie and strap around waist	Onesie and strap around waist	Onesie with long legs including foot covering and strap for ankle

**The battery life of GENEActiv is determined by the measurement frequency selected to record. For example, recording at 100Hz records for 7 days and 10Hz records for 45 days (activinsights.com)*

+The battery life of ActiGraph is determined by the measurement frequency selected to record.

#Information correct as at 7th February 2018
